# Machine learning algorithms to predict feeding practices during diarrheal disease and its determinants among under-five children in East Africa

**DOI:** 10.3389/fpubh.2025.1513922

**Published:** 2025-07-23

**Authors:** Tirualem Zeleke Yehuala, Nebebe Demis Baykemagn, Bewuketu Terefe

**Affiliations:** ^1^Department Health informatics, Institute of Public Health, College of Medicine and Health Sciences, University of Gondar, Gondar, Ethiopia; ^2^Department of Community Health Nursing, School of Nursing, College of Medicine and Health Sciences, University of Gondar, Gondar, Ethiopia

**Keywords:** feeding practice, diarrhea, determinants, East Africa, machine learning model, prediction

## Abstract

**Background:**

Diarrhea is the leading cause of childhood malnutrition. Although replacement, continued feeding, and increasing appropriate fluid at home during diarrhea episodes are the cornerstones of treatment packages, food and fluid restrictions are common during diarrheal illnesses in Africa. To fill the methodological and current evidence gaps, this study aimed to build models and predict determinants to increase feeding practices of children in East Africa during diarrheal outbreaks.

**Methods:**

We used the most recent demographic and health survey (DHS) statistics from 12 East African nations collected between 2012 and 2023. The analyses included a total weighted sample of 20,059 children aged 5 years. Python software was utilized for data processing and machine learning model building. We employed four ML algorithms, such as Random Forest (RF), Decision Tree (DT), XGB (Extreme Gradient Boosting), and Logistic Regression (LR). In this work, we evaluated the predictive models' performance using performance assessment criteria such as accuracy, precision, recall, and the AUC curve.

**Results:**

In this study, 20,059 children aged 5 years were used in the final analysis. Among the proposed machine learning models, random forest performed best overall in the proposed classifier, with an accuracy of 97.86%, precision of 98%, recall of 77%, F-measure of 86%, and AUC curve of 97%. The most significant determinants of increasing feeding practice were richest household, faculty delivery, use of modern contraception method, the number of children 3–5, women's employment status, maternal age is 25–34, having media exposure, and health-seeking decisions made by mothers were associated positively, whereas not using contraception, home delivery, the total number of children is large, and the sex of the household was male, which was associated negatively with feeding practice during diarrhea in East Africa.

**Conclusion:**

Machine learning (ML) algorithms have provided valuable insights into the complex factors influencing feeding practices during diarrheal disease in under-five children in East Africa. During diarrhea, only 11 of the 100 children received acceptable child feeding practices. More than one-third of the patients received less than usual or nothing. Reducing diarrhea-related child mortality by improving diarrhea management practices is recommended, particularly focusing on the identified aspects.

## Introduction

Diarrhea is defined as three or more loose or watery stools within 24 h or an increase in a person's daily stool fluidity, frequency, or volume compared to what is considered normal (1, 2). Diarrhea, like other infectious illnesses, can be caused by inadequate hygiene, contaminated food, and polluted drinking water passed from person to person (2).

Most recent WHO factsheet (2024) on diarrhea states that diarrhea is the third leading cause of death in children under the age of five (3).

Since 2018, there have been almost 1.7 billion instances of childhood diarrhea per year, and an estimated 5.3 million children under the age of five have died, largely from preventable causes, with nearly half of the deaths occurring within the first month of life (2, 4). Sub-Saharan Africa (SSA) and Southern Asia account for more than 80% of all under-five deaths (5). In Africa, diarrhea is the leading cause of illness among young children, accounting for approximately half of all fatalities in this age group (6).

If not treated properly, childhood diarrhea can result in reduced macronutrient and micronutrient intake, further episodes of infectious disease, malnutrition, impaired physical growth, and impaired cognitive development (7, 8). Optimal feeding practices (both liquid and solid foods) during childhood illnesses are among the most effective global strategies for the integrated management of childhood illnesses, emphasizing the need to increase fluid intake while maintaining feeding during convalescence (9, 10).

The existing literature on rehydration management for Acute Watery Diarrhea (AWD) emphasizes the importance of Oral Rehydration Therapy (ORT) as the primary treatment, particularly in resource-limited settings (11). Oral Rehydration Solution (ORS) is a mixture of electrolytes and sugar that is used to replace lost electrolytes such as potassium, sodium, chloride, and bicarbonate because of diarrhea (12). ORS is usually prescribed for children under five who present with some acute watery diarrhea with no or some dehydration (13). Studies have demonstrated that oral glucose-electrolyte solutions effectively reduce the need for intravenous therapy, especially in cholera patients. However, alternative rehydration methods, such as nasogastric (NG) rehydration, have been found to be equally effective for moderate-to-severe dehydration (14). Intravenous (IV) fluid therapy remains essential for severe dehydration, with the literature distinguishing between repletion and maintenance therapy (the latter aimed at sustaining fluid balance). Additionally, research has explored intraosseous (IO), intraperitoneal (IP), and subcutaneous rehydration, though further evidence is needed to validate their effectiveness (15). A parents and caregivers continue to feed their children through a diarrheal episode, the repercussions of the disease will be less severe and the child's death will be avoided (2, 16, 17).

Children's nutritional condition can occasionally deteriorate during or after illness if they do not receive extra food. Low appetite due to diarrhea in children can contribute to the continuation of the illness and stunting cycle (18). Regardless of the multifaceted benefits of continuous feeding of children with diarrheal disease, some traditional beliefs and practices, such as partial food restrictions, cessation of breast milk, giving foods in a specific composition and amount, and restriction of vegetables and fruits, continue to contribute to inappropriate feeding practices during diarrhea among children (19–21). Furthermore, previous research has found that occupation (17), wealth index (22), maternal age (17, 23), maternal education (22, 24), number of under-five children, and knowledge of child feeding practices (17, 25) are all related to optimal child feeding practices. Evidence suggests that only 35% of under-five children in the region receive sufficient fluid replacement during diarrheal episodes (26). Currently, there is a methodological gap in the previous literatures based on recent data from East African countries (27, 28). Classical statistical methods have limitations in capturing complex and nonlinear relationships between predictors and outcomes. Machine learning algorithms (ML) are typically designed to make accurate predictions by learning from data rather than making prior assumptions. By utilizing ML algorithms, this study addressed uncovered-hidden patterns and interactions within the data, leading to more accurate predictions and a great understanding of the important determinates feeding practice during diarrheal disease. The data-driven insights can inform evidence-based decision-making and resource allocation for treatment related service and policies.

## Methods

### Data sources and sampling procedures

In this study we analyzed the most recent Demographic and Health Survey (DHS) data from 12 East African nations (Burundi, Ethiopia, Comoros, Kenya, Madagascar, Malawi, Mozambique, Rwanda, Tanzania, Uganda, Zimbabwe, and Zambia) collected between 2012 and 2023 to determine the practice of receiving multiple micronutrient powders and the factors that influence it in East Africa. These current datasets were combined to investigate the prevalence and associated determinants of various solid and liquid food feeding practices during diarrhea among children under 5 years of age in Eastern Africa.

The DHS surveys were collected over a 5-year cycle in low- and middle-income countries using structured, pretested, and validated questionnaires. The DHS surveys used the same standard sampling, questionnaires, data collection, and coding procedures, allowing for multi-country analysis. A stratified two-stage cluster sampling was used for this study. At the first stage, a stratified samples of EAs were selected with probability proportional to size in each country of the most recent national census. At the second stage, after a complete household listing was conducted in each of the selected EAs, fixed number of households were selected by equal probability systematic sampling in the selected EAs in each country (29).

### Population, and eligibility criteria

Interviews were conducted with target demographics (women aged 15–49 and males aged 15–64) in selected houses. This study included all under five children who had experienced diarrhea in the 2 weeks preceding the most recent DHS in 12 East African nations. According to the DHS Statistics Guide, missing values and “don't know” are omitted from the numerator (assumes did not consume or receive). Any missing data for any outcome variable was managed using various missing data management procedures in accordance with the instructions of the DHS statistics guide (30). A total of 20,059 under five children were involved in the study (Table 1).

**Table 1:** Countries, sample size, and survey year of Demographic and Health Surveys included in the analysis for 12 East African countries.

**Country**	**Survey year**	**Sample size (weighted)**	**Frequency (weighted)**
Burundi	2016/17	2,861	14.26
Ethiopia	2016	1,209	6.03
Kenya	2022	2,380	11.86
Comoros	2012	480	2.39
Madagascar	2021	1,048	5.22
Malawi	2016/17	3,540	17.65
Mozambique	2023	1,126	5.62
Rwanda	2019/20	1,129	5.63
Tanzania	2022	1,109	5.53
Uganda	2016	2,784	13.88
Zambia	2018	1,389	6.92
Zimbabwe	2015	1,005	5.01

### Study design and study period

This study adopted a cross-sectional study design for further analysis of DHS, which was conducted from 2012 to 2023. In this study, we design and develop the machine learning model. This involves selecting the appropriate algorithm, pre-processing the data, and engineering features. Finally, we develop a predictive model that predicts the feeding practice and identify it determinants to increase feeding practice during diarrheal disease among under five children.

### Data analysis procedure

Stata version 17 and Python software were used to extract, recode, and analyze the data. Weighting was used throughout the study to ensure representativeness and non-response rates as well as to obtain a suitable statistical estimate (robust standard error) (31). We employed Python software with the most crucial packages are Panda, Scikit Learn, Imblearn, Numpy, and Matplotlib for data preparation, model planning, model construction, model assessment, model deployment, and rule generation in this study. In conclusion, we create a predictive model that predicts the determinants to increase the feeding practice during diarrheal disease among under five children.

### Variables of the study

#### The outcome variable

The outcome variable of this study was feeding practice during diarrheal. Then, the outcome variable was categorized as “less than usual” (somewhat less, much less, or none) and “more than usual.” The first category less than usual was assigned a value of “0,” while the second category (more than usual) was assigned a value of “1.” This classification and analysis were conducted according to the guidelines in the DHS Statistics Handbook (30, 32).

#### Independent variables

Various maternal and child-related factors were included. This included maternal age, educational status, type of place of residence, marital status, household wealth index, current employment status, mass media exposure, ANC follow-up, place of delivery, PNC check-up, persons usually deciding on mothers' health seeking, number of health visits and total children born, under five children, contraceptive utilizations, age of the child, sex of the child, size at birth, twin status, birth order, sex of the household head, and countries.

### Ethical considerations and data set access

The study was conducted after obtaining a permission letter from www.dhsprogram.com on an online request to access East African DHS data after reviewing the submitted brief descriptions of the survey to the DHS program. The datasets were treated with the utmost confidence. This study was done based on secondary data from East Africa DHS. Issues related to informed consent, confidentiality, anonymity, and privacy of the study participants are already done ethically by the DHS office. We did not manipulate and apply the micro data other than in this study. There was no patient or public involvement in this study, and no ethical clearance was required.

### Data pre- processing

#### Data cleaning

We employed data cleaning, which typically includes noise, imbalanced outcome variables, and missing values. Our dataset contains some features that have missing values for data that is categorical data type. We used the mode imputation method to fill in the missing values. In this study among 20,059 records had 114 missing records for critical features, comprising nearly 0.56%. Features like such as place of residence: 0.0061%, marital status: 0.003%, wealth index: 0.001% contraceptive utilizations: 0.02%, age of the child: 0.007, sex of the child: 0.0031, size at birth: 0.053, twin status: 0.046, birth order: 0.3578.

#### Data transformation and data integration

In this study, we utilized data transformation, which involves converting the data from one format to another that is suitable for analysis, which involves transforming data types, scaling data, and normalizing data. In this study, the data set has different data formats, like categorical and numeric features. One-hot encoding is a technique employed to convert categorical to numerical data. We integrate the data from 12 East African countries to ensure consistency and standardization; it is suitable for training machine learning models.

#### Data discretization

In this study we used data discretization for converting continuous data (numerical values) into discrete categories or intervals (33). In this study, data discretization was used, which limits the impact of outliers and reduces noise by transforming continuous variables into categorical features according to DHS guidelines to make the data easier to understand and analyze.

#### Feature selection

**Figure 1:**
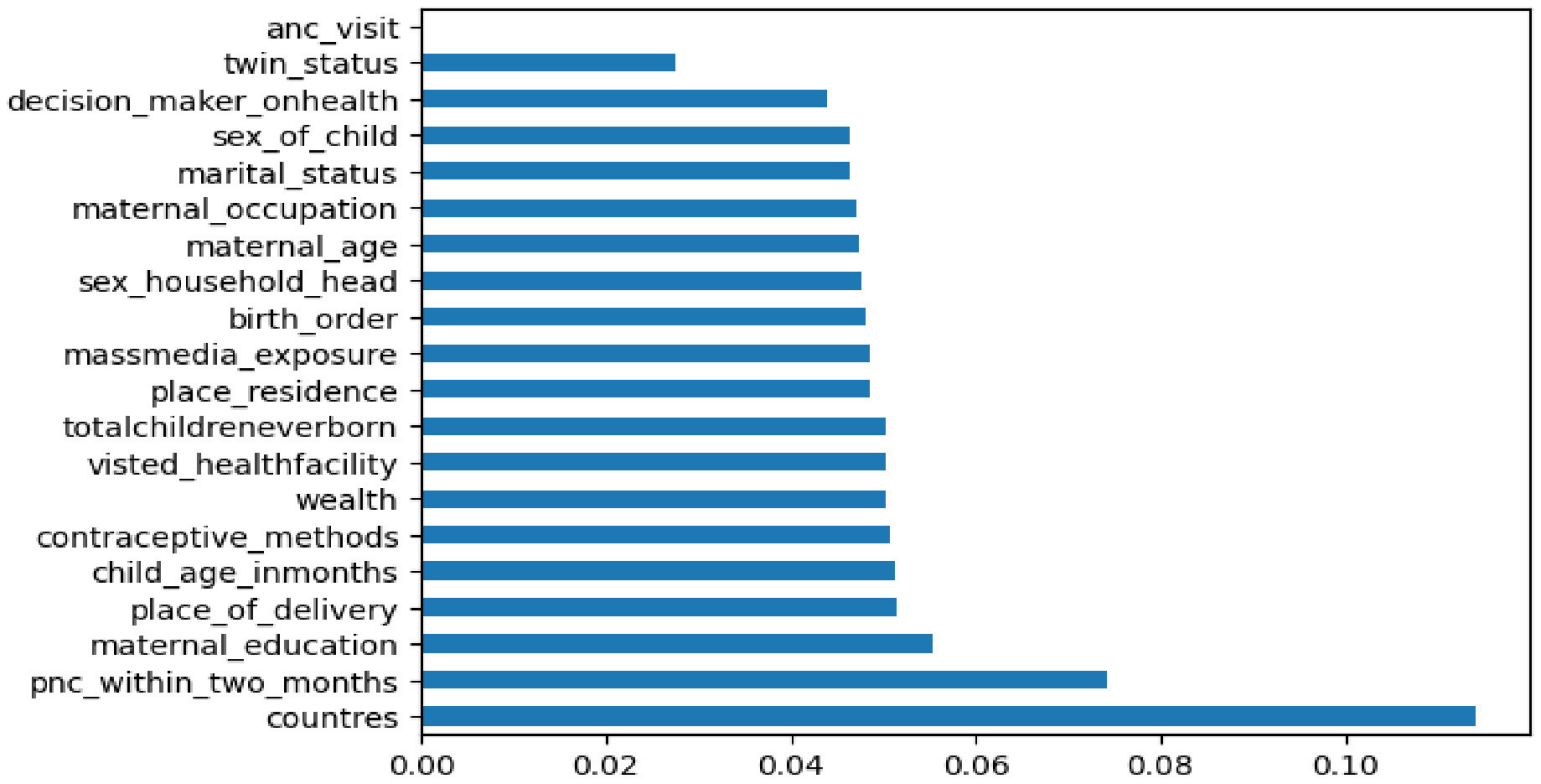
Important determinates selected by RFE.

In this study we utilized feature selection techniques to removing irrelevant or redundant the number of features during developing a predictive model. The feature selection methods were used in data preprocessing to achieve efficient data reduction. In this study, we employed recursive feature elimination (RFE) to identify the most relevant variables for predicting increased feeding practices during diarrheal disease. Recursive Feature Elimination (RFE) is a feature selection technique that systematically identifies the most relevant predictors for a model by iteratively removing less important features. It begins by training a model on all available features and evaluating their importance using metrics like coefficients or feature importance scores. The least significant features are then eliminated, and the process is repeated until the desired number of predictor's remains. RFE enhances model performance by focusing on essential features, reduces overfitting by excluding irrelevant data, and improves interpretability by simplifying the model. In this study the dataset contains 41 features or variables. To select the most important features, we employed fast recursive feature elimination. This approach offers flexibility in controlling the number of features retained and can effectively handle correlated features. Maternal age, educational status, type of place of residence, marital status, household wealth index, current employment status, mass media exposure, ANC follow-up, place of delivery, PNC check-up, persons usually deciding on mothers' health seeking, number of health visits and total children born, under five children, contraceptive utilizations, age of the child, sex of the child, size at birth, twin status, birth order, sex of the household head, and countries were selected by (fast recursive elimination) RFE, and these determinants were used for model building as shown in Figure 1.

#### Class balancing

To develop the model the training data was imbalance. In this study we employed Synthetic Minority Oversampling Technique (SMOTE) this technique produce additional observation. Prior to smote balancing, less than usual and same as usual was 14,282 (89%), while the prevalence more than the usual was 1,765 (11%). We obtained a balanced sample of children who less than the usual and same as usual with counts 14,282 and more than the usual with count 14,282.

#### Data splitting

Data splitting is an important step in machine learning to ensure the proper evaluation of a model's performance. The goal is to divide the available dataset into subsets to train and test the model. This helps in assessing how well the model generalizes to unseen data and prevents over fitting to the training set. In this study, we utilized a simple holdout method like 80% (16,047 case) training data for model building and 20% (4,012 case) testing for model performance.

#### Model selection

The predicted variable in this study was binary prediction, which refers to predicting one of two possible classes (e.g., yes/no, true/false, 1/0). In this study, we utilized four supervised machine learning models, such as random forest (RF), decision tree (DT), logistic regression (LR), and extreme gradient boosting (XGB). The algorithms were chosen in accordance with previous research that used machine-learning methods to classify tasks (34, 35).

#### Model interpretation based on SHAP values

A feature's SHAP value indicates the extent to which its value influences the discrepancy between the average model output and the anticipated output of the model (36). In this study we used SHAP values for model interpretation, since features that push the prediction higher compared to the base value have positive SHAP values, while features that push the prediction lower have negative values.

## Results

### Socio demographic characteristics of the study participant

In this study, 20,059 children who experienced diarrhea and whose age was from 6 to 59 months old were enrolled in East African countries. Approximately half of them, 9,181 (45.77%), were between 25 and 34 years old within the reproductive age range. Regarding marital status, the majority of the mothers, 13,247 (66.04%), were married. With respect to place of residence types, 15,743 (78.48%), educational status 10,514 (52.42%), wealth index 4,928 (24.57%), place of delivery 16,256 (81.04%), and ANC follow-up 19,174 (95.59%) mothers were from rural, primary educational status, poorest households, institutional delivery, and had at least one ANC follow-up during their pregnancies. Similarly, about 10,655 (53.12%) and 12,948 (64.55%) women had no experience of at least one mass media exposure (listening to radio, watching television, reading magazines or newspapers) and were employed, respectively. About 15,770 (78.62%) women were unable to access healthcare services by themselves when they needed it. Children-related variables, such as weight, age, sex, and birth order, were 8,950 (48.16%), 4,940 (48.59%), 10,617 (52.93%), and 7,393 (36.86%) of children were average weighted, 12–35 months old, male, and 2nd or 3rd birth orders, respectively. Similarly, approximately 16,668 (83.10%) of fewer than three children were found in each household. The majority of 19,516 (97.29%) children were born single, as shown in Table 2.

**Table 2:** Maternal and child-related socio demographic characteristics of respondent's feeding practice during diarrhea among reproductive-age women in East Africa (*n* = 20, 059): based on the recent East African countries DHS data.

**Variables**	**Categories**	**Less than usual**	**More than usual**	***P*-values**
Age in years	15–24	6,227 (37.7%)	793 (36.39%)	X^2^ = 2.9e+04, *p*-value = 0.000
	25–34	7,933 (45.48%s)	978 (44.82%)	
	35–49	3,284 (28.83%)	408 (18.73%)	
Residence	Urban	3,857 (22.11%)	544 (24.97%)	X^2^ = 8.5e+03, *p*-value = 0.000
	Rural	13,587 (77.89%)	1,637 (75.03%)	
Mothers' educational status	No education	4,055 (23.25%)	403 (18.49%)	X^2^ = 86.4104, *p*-value = 0.000
	Primary	9,052 (51.89%)	1,140 (18.49)	
	Secondary and higher	4,337 (24.86%)	636 (29.19%)	
Mothers employed	No	6,328 (36.28%)	740 (36.26%)	X^2^ = 86.4104, *p*-value = 0.000
	Yes	11,116 (63.72%)	1,389 (63.74%)	
Wealth index	Poor	8,561 (49.08%)	869 (39.88%)	X^2^ = 2.2e+03, *p*-value = 0.000
	Middle	3,287 (18.84%)	435 (19.96%)	
	Rich	5,596 (32.08%)	875 (40.16%)	
Mass media exposure	No	9,505 (54.48%)	1,112 (51.03%)	X^2^ = 638.3930, *p*-value = 0.000
	Yes	7,941 (45.52%)	1,067 (48.97%)	
ANC follow-up's	No	585 (37.15%)	300 (38%)	X^2^ = 417.876, *p*-value = 0.000
	Yes	10,161 (62.85%)	600, 13 (62%)	
Place of delivery	Home	3,465 (19.86%)	346 (15.88%)	X^2^ = 1.5e+03, *p*-value = 0.000
	Health facility	13,979 (80.14%)	1,833 (84.12%)	
Total number of children ever born	0–2	9,101 (52.17%)	1,184 (54.34%)	X^2^ = 9.6e+03, *p*-value = 0.000
	3–6 and above	8,343 (47.83%)	995 (45.66%)	
Contraceptive method types	No methods	9,268 (53.13%)	1,028 (47.18%)	X^2^ = 1.6e+03, *p*-value = 0.000
	Traditional	479 (2.75%)	74 (3.40%)	
	Modern	7,697 (44.12%)	1,077 (49.43%)	
Number of health visits in the past 12 months	Once	3,918 (21.62%)	379 (18.22%)	X^2^ = 3.7e+06, *p*-value = 0.000
	More than one	12,751 (78.38%)	379 (18.22%)	
Marital status	Never married	994 (5.70%)	128 (8.87%)	X^2^ = 1.1e+045, *p*-value = 0.000
	Married	11,621 (66.62%)	1,476 (67.74%)	
	Divorced/widowed	4,829 (27.68%)	575 (26.39%)	
Size of the child	Small	7,367 (47.22%)	983 (45.11%)	X^2^ = 4.3e+03, *p*-value = 0.000
	Average	6,845 (39.24%)	816 (37.45%)	
	Large	3,234 (18.54%)	380 (17.44%)	
Sex of the child	Male	9,101 (52.17%)	1,184 (54.34%)	X^2^ = 1.6e+541, *p*-value = 0.000
	Female	8,343 (47.83%)	995 (45.66%)	
Twin status	Single	16,978 (97.33%)	2,118 (97.20%)	X^2^ = 7.9e+03, *p*-value = 0.000
	Multiple	466 (2.67%)	61 (2.80%)	
Sex of the household head	Male	13,196 (76.65%)	1,637 (75.13%)	X^2^ = 5.4e+23, *p*-value = 0.000
	Female	4,248 (24.35%)	542 (24.87%)	

### Prevalence of feeding practice during diarrhea among under five children in East Africa

In this study, a higher proportion of mothers (53.54) (95% CI, 52.86, 54.24) had provided the foods and liquids just the same as usual. In contrast, about 34.85 (34.19, 35.5) had practiced less than usual while their children had experienced diarrhea.

### Comparing Supervised ML Classifier

The goal of this study was to build a predictive model and identify determinants to increasing feeding practice during diarrhea disease among under five children. We utilized four supervised machine learning algorithms, such as random forest, decision tree (DT), logistic regression (LR), and XGB (Extreme Gradient Boosting). The experiments were done with the same testing parameters. In this study, the predictive performance was evaluated based on the five performance measures (accuracy, precision, F-measure, AUC curve, and recall). After comparing ML models, random forest emerged as the best predictive model, with an accuracy performance of 97.86%, precision of 98%, recall of 77%, F-measure of 86%, and AUC curve of 97%.

**Figure 2:**
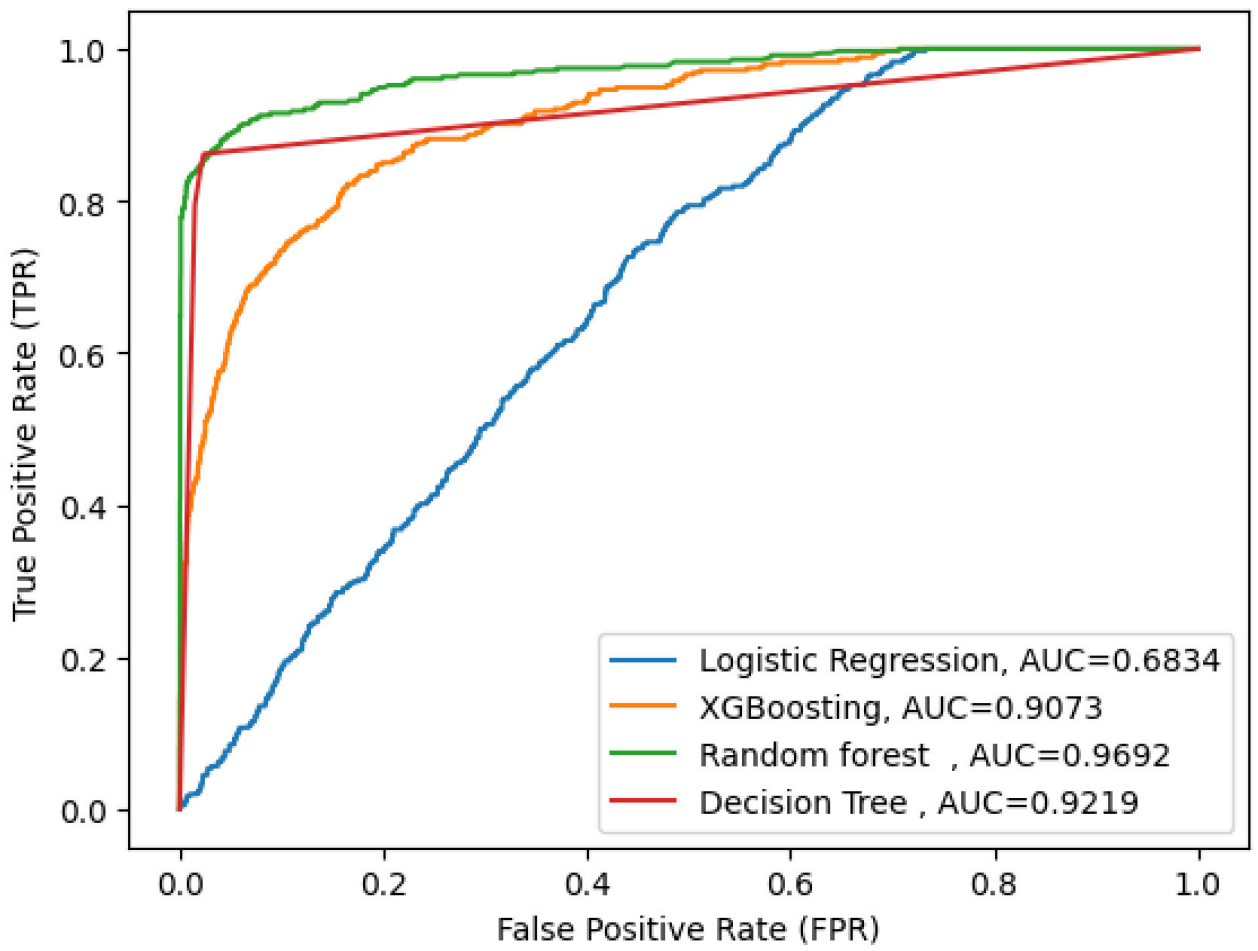
AUC curve for selected machine learning model.

In addition, random forests had high specificity (87%) and sensitivity (77%). The true positive rate of the random forest was 96%, the false positive rate was 3.9%, and the AUC curve was high, 97%. Moreover, decision tree (DT) has an accuracy of 96.79%, precision of 83%, recall of 79%, F-measure of 81%, and an AUC curve of 92%. Among the proposed models, logistic regression was the least one with an accuracy of 88%, precision of 58%, F-measure of 19%, recall of 71%, and an AUC score of 68%, since the logistic regression model was highly mis_predicted, as shown in Figure 2 and Table 3.

**Table 3:** Performance comparison of machine learning models using accuracy, precision, recall, and F-measure.

**Machine learning models**	**Dataset**	**Accuracy**	**AUC**	**Precision**	**Recall (sensitivity)**	**F1-score**	**95 % confidence interval**
Decision tree	Balanced (%)	96.79	92	83	79	81	[0.839, 0.883]
Random forest classifier	Balanced (%)	**97.86**	**97**	**98**	**77**	**86**	[0.925, 0.972]
Extreme gradient boosting	Balanced (%)	92.69	90.7	91	20	34	[0.887, 0.925]
Logistic regression	Balanced (%)	88	68	71	15	19	[0.843, 0.895]

To evaluate the uncertainty of our findings, we used a 95% confidence interval (CI), which indicates that we have a 95% chance that the true value falls within the computed range. In Table 3 shown for random forest model between 92.5 and 97.2% is the true accuracy with 95% confidence.

**Figure 3:**
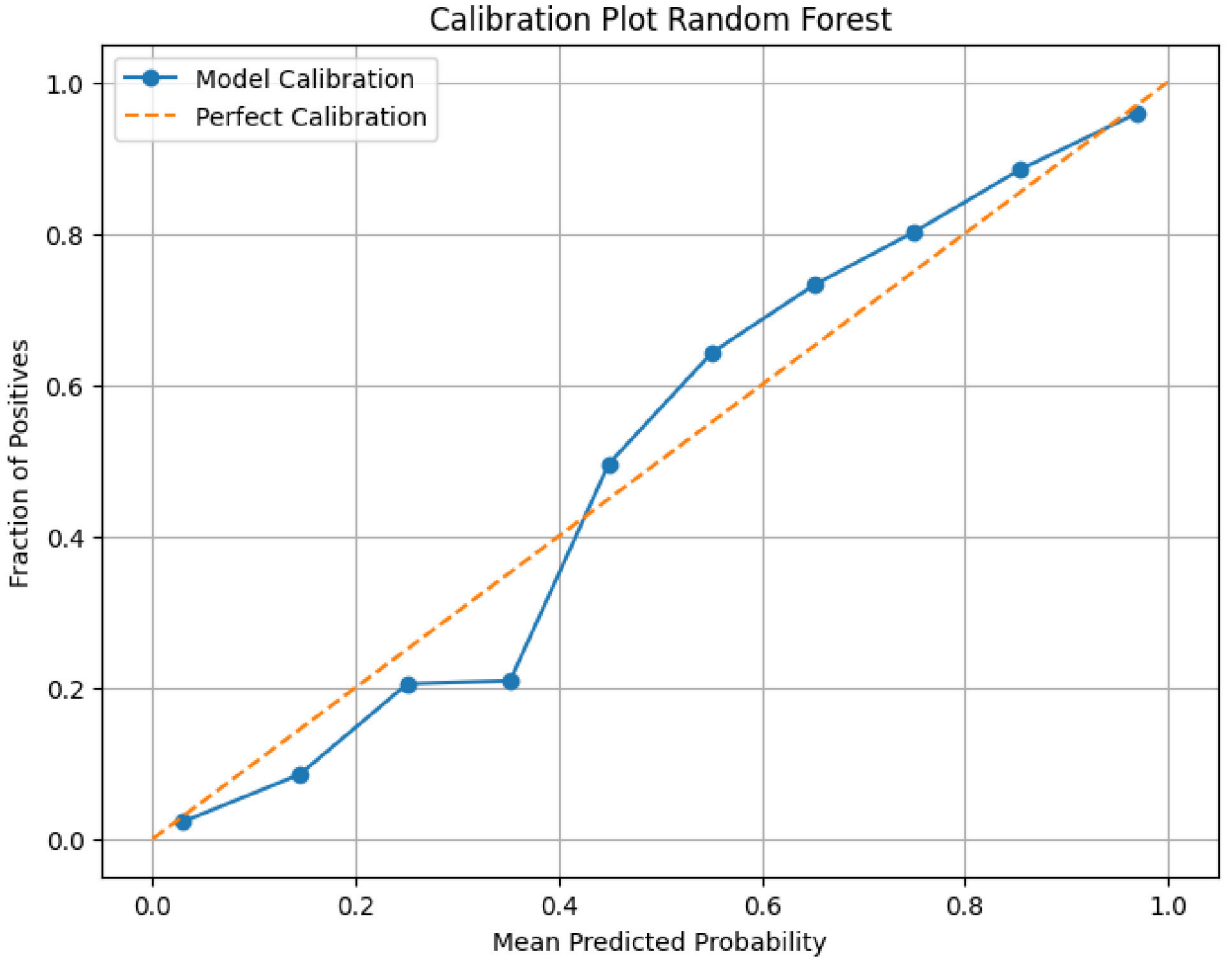
Calibration plot for the Random Forest model.

Additionally in this study we utilized a calibration plot to evaluate how well the predicted probabilities of a model align with actual outcomes. It's particularly useful for probabilistic classifiers championing random forest models to assess whether the predicted probabilities correspond to the true outcomes as shown in Figure 3.

### Model interpretation

**Figure 4:**
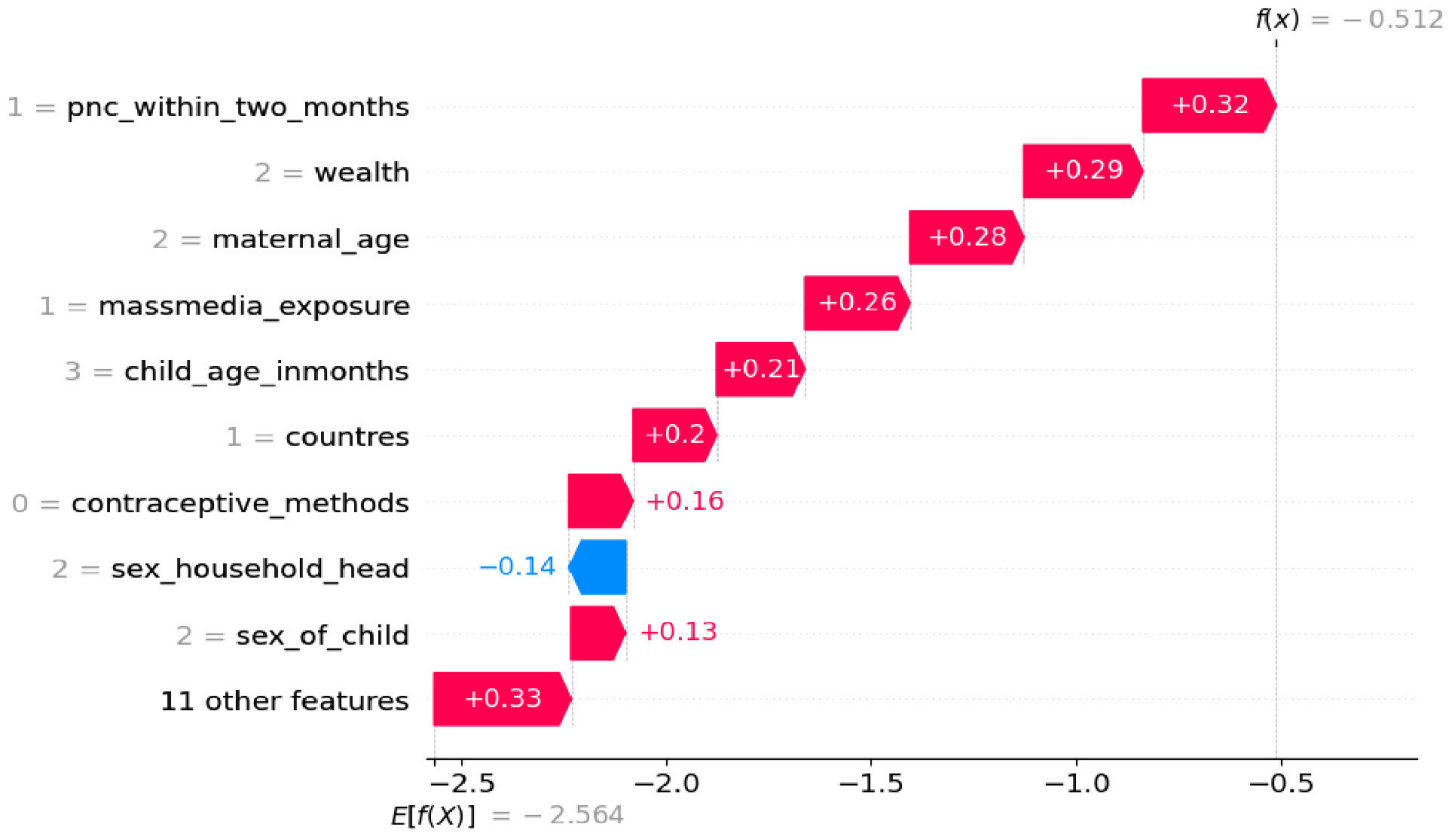
Waterfall plot displaying prediction of the zero index observation.

In this study we used SHAP values for model interpretation, since features that push the prediction higher compared to the base value have positive SHAP values, while features that push the prediction lower have negative values. Therefore, for the first observation, the combination of the positive contributions (in red) and the negative contributions (in blue) moves the expected value output to the final model output [f_(x)_ = −2.183], classified as a negative class less than usual. In this case, features like being 2nd or 3rd birth order, faculty delivery, being female, using modern contraception methods, the number of children 3–5, women's employment status, the wealth status being rich, maternal age being 25–34, having media exposure, and health seeking decisions made by mothers and other features impacting children feeding more than usual during diarrheal disease show in Figure 4.

**Figure 5:**
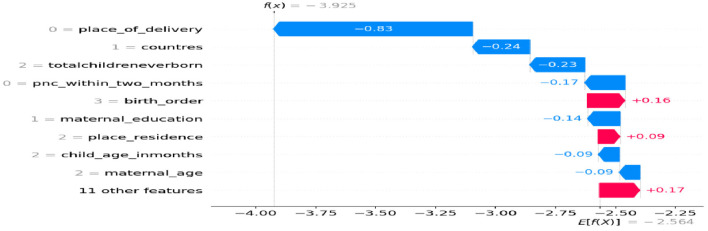
Waterfall plot displaying prediction of the 1st observation.

However, having no PNC checkup within 2 months, delivery at home, a number of birth orders above 5, a mother is not educated, who lives in rural areas, no at least one ANC, and other features has an impact of each predictor (less than usual) on children feeding during diarrheal disease (Figure 5).

### Sensitivity analysis

**Figure 6:**
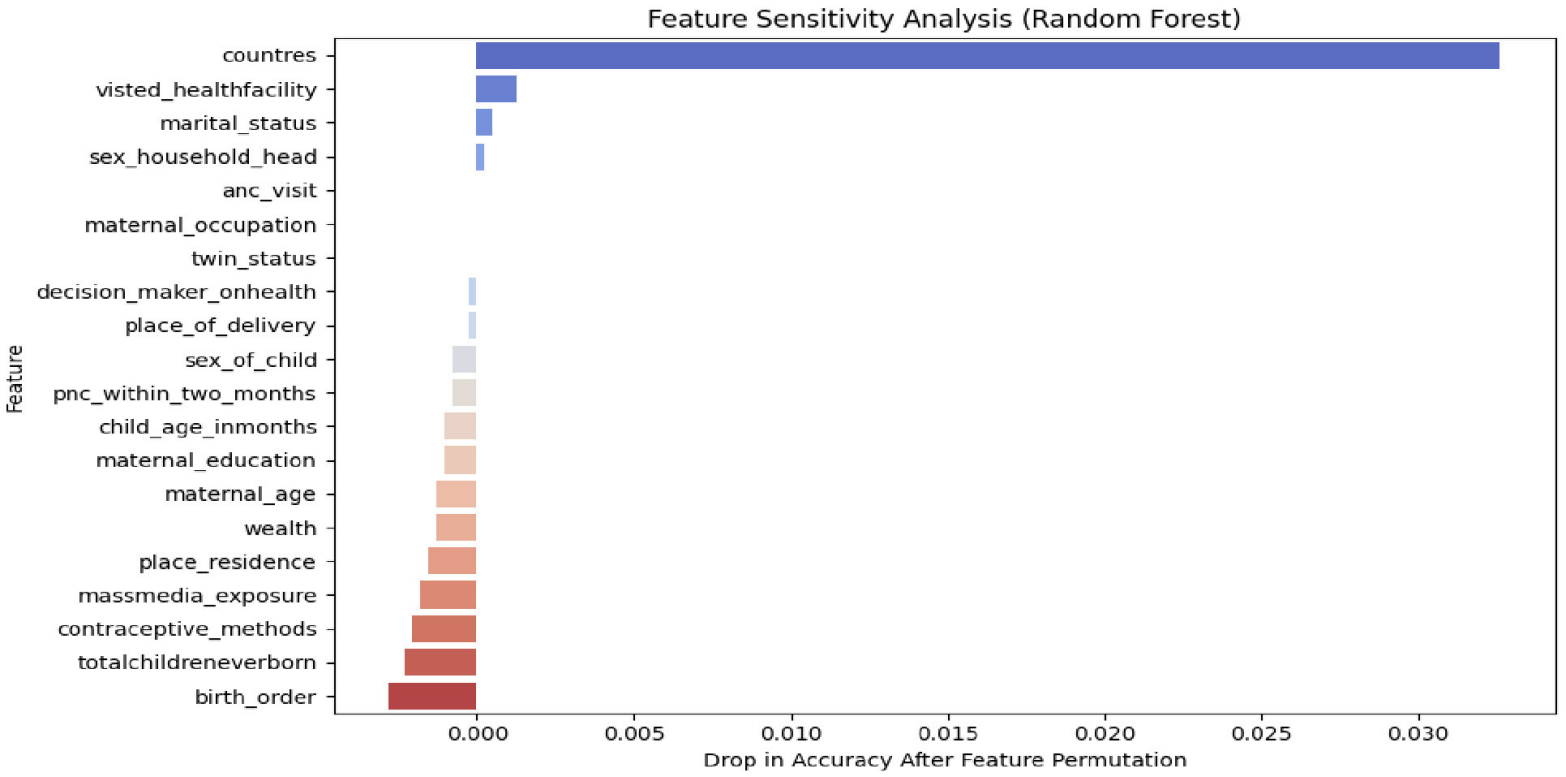
Sensitivity analysis with SHAP values.

In this study, we employed sensitivity analysis to understand which features have the greatest influence on the model's predictions and to assess its stability and robustness in the face of changes. Specifically, we utilized global sensitivity analysis as showed Figure 6, which involves evaluating the overall contribution of each feature to the model's predictions and examining how they interact with one another. Features toward the top are the most significant (i.e., the model is most sensitive to them), as Figure 6 illustrates. Red points that are primarily on the right show that the model's prediction is increased by high feature values. We developed the models on the ≤ 2020 dataset, and then temporally validated them on the ≥2021 dataset. Among the proposed algorithm random forests had high Accuracy with 90.39%, AUC 92%, precision 93.6% and recall 80.13% as shown in Table 4.

**Table 4:** Sensitivity analysis of the proposed machine learning algorithms: internal validation on the 2020 dataset and temporal validation on the 2021 dataset.

**Machine learning models**	**Accuracy (%)**	**AUC (%)**	**Precision (%)**	**Recall (%)**
Decision Tree	89.79	90	84	69
Random forest classifier	**90.39**	**92**	**93.6**	**80.13**
Extreme gradient boosting	90	89.9	90.6	78
Logistic regression	69.4	67	59	52

## Discussion

According to the World Health Organization's recommendations for continued feeding during diarrhea, as the nutritional complications of diarrhea have become more widely recognized (37–39) more attention has been focused on the appropriate dietary management of this illness (40, 41). To tailor different intervention measures to reduce diarrhea-related mortality among under-five children in East Africa. This study aimed to investigate the current magnitude of appropriate feeding practices and identify the determinants (associated factors) to increase feeding practice among under-five children with diarrheal disease. For this objective, four machine learning models were trained on balanced training data using train-test split. Accuracy, AUC score, precision, recall, and F1 score were used to compare the performance of classifier models. Random forest emerged as the best predictive model, with an accuracy performance of 97.86%, precision 98%, recall 77%, F-measure 86%, and AUC curve 97%. To date, no studies have been conducted utilizing ML algorithms to predict determinants of increasing feeding practice during diarrhea among under-five children. However, the study conducted in Ethiopia, titled Comparative Analysis of Machine Learning Algorithms for Predicting Diarrhea Among Children Under Five in Ethiopia: Evidence from the 2016 EDHS, found that the Random Forest algorithm performed the best, with an accuracy of 93.2%, sensitivity of 98.4%, specificity of 85.5%, and an AUC of 0.916 (42). This study discovered the prevalence of feeding practice: only 11.60% of children under five with diarrhea received more appropriate food during the diarrheal episode than usual. On the other hand, ~88.4% of them received less than usual or did not take anything during their illness, and the rest are taken as usual. According to the data, a considerable percentage of children in Eastern Africa do not receive appropriate food and fluid during their diarrheal episodes, indicating that this issue is a high priority (23, 43).

In the feature selection, maternal age, children's age, maternal employment, maternal education, visit health facility, place of delivery, media exposure, wealth index, having at least one ANC, mother's health seeking for decision-making on children's health, and child's age were extremely associated with the outcome variable.

Employed women had a 16% of feeding their children more than normal, rather than less than usual, when all other characteristics remained constant. Similarly, when other variables were held constant, mothers from poorer, middle, richer, and richest households had a higher risk of feeding their children more than usual than women from the poorest household wealth index by 24, 45, 67, and 28%, respectively, rather than feeding less than usual. The factor linked with proper child feeding practices in this study was women's employment status, with those with current employment having a higher chance of having adequate child feeding practices than their counterparts. Studies from Nepal and SSA have found similar associations (23, 44).

This could be related to the positive impact of women's employment on household wealth status since working women typically have financial means that allow them to buy appropriate food and feed their children. Their social connections can help improve their knowledge and attitudes regarding eating practices.

Similarly, in earlier studies (22, 23, 45), women with a higher wealth index had a higher likelihood of having a child with acceptable or more than normal feeding practice compared to women with the lowest wealth index. Families with greater financial resources tend to have better access to healthcare, education, and nutritional knowledge, which can lead to appropriate feeding practices during diarrheal episodes. The study conducted sub-Saharan Africa found that children from wealthier households were more likely to receive adequate food and fluids during diarrhea compared to those from lower-income families (46). This is apparent because, in comparison to their counterparts, women with high incomes have no budget limits to purchase various dietary items and fluids for the treatment of diarrhea.

Holding all other variables constant, mothers with 12–35-month-old children and 36–59-month-old children had a higher relative risk ratio tendency to feed their children more than the usual rather than less than the usual, as compared to 6–11-month-old children, respectively. Similarly, child age was reported as a predictor variable in research conducted in five Asian nations (47), Tanzania (48), and northern Ethiopia. Mothers may be uninformed or they may need to continue breastfeeding their children rather than feeding them more than normal. If these children are breastfed properly, they may have a lower risk of experiencing diarrhea. This may provide opportunities for health program planners to focus on the feeding of younger children. The problem of appropriate feeding of children aged 6–11 months is supported by findings from a previous study conducted in Ethiopia, which revealed a high prevalence of stunting (43%) among children aged 6–11 months, there is a need to place more emphasis on feeding of children aged 6–11 months (49). Additionally studies show that the prevalence of appropriate feeding practices during diarrheal illness among children aged 6–23 months is alarmingly low, particularly in Sub-Saharan Africa. Factors such as maternal education, media exposure, vaccination, and regional differences significantly influence these practices (17).

Holding all other variables constant, having at least one ANC visit during pregnancy and giving birth in a health facility increases the relative chance of feeding more than normal rather than feeding less than usual to 1.49 and 1.19 (increases by 49 and 19%, respectively). Similarly, when all other dependent variables were maintained constant, not using any type of contraception reduced the chance of feeding more than normal rather than feeding less than usual to 0.89 (an 11% reduction). Studies from Ethiopia and SSA have found a similar link between these and other maternal health services (10, 50, 51). Possible arguments include the fact that these services are an excellent point of contact for providing counseling on child feeding practices, in addition to maternity and childcare. Mothers who have ANC and deliver births in a health facility may obtain information, education, and counseling about appropriate infant feeding practices for normal child growth and during an illness, such as diarrhea, from health professionals. Similarly, mothers who use contraception will have a better understanding of feeding practices and fewer children. These interns allowed them to feed their children more than usual.

In this studies emphasize that appropriate practices, such as continuing breastfeeding, having ANC follow up, and offering nutrient-rich foods, but are often underutilized due to cultural beliefs, misinformation, and socioeconomic factors. Harmful practices like unclean food or fluids exacerbate the risk of malnutrition and prolonged illness. Public health strategies, such as educational programs for mothers or caregivers, promoting access to safe water and sanitation, and vaccination have shown promise in addressing these gaps. These strategies can significantly reduce the prevalence of diarrheal diseases and improve child health outcomes.

## Conclusions

In this study, the prevalence of adequate child-feeding practices was found to be quite low in East Africa. In this study, maternal employment status, antenatal care visits, place of delivery, contraceptive use, and child age were all independently related to optimal feeding practices during a diarrheal episode in children under the age of five in East Africa. Thus, intensified intervention programmers and stakeholders working on child health should concentrate on these determinants in order to minimize child death and morbidity and achieve the region's sustainable development goals. Particularly, supporting maternal-related health services and making use of opportunities will have a greater impact on increasing mothers' feeding practices for their infants.

### Strength and limitations

This study aimed to predict feeding practices during diarrheal diseases and identify key predictors. It also utilized the most recent DHS dataset from East African countries, which, due to its large sample size, improves the generalizability of the results. However, the study has certain limitations. Since the data were collected based on mothers' memories, there is a risk of recall bias, and some details may have been overlooked. Additionally, while the study employed mode imputation techniques to handle missing data, these methods have notable limitations, particularly when dealing with high frequencies of missing data. However, the number of missing values in this study was small.

## Data Availability

Publicly available datasets were analyzed in this study. This data can be found here: www.dhsprogram.com.
